# Diagnostic accuracy of fine needle aspiration biopsy in pediatric small round cell tumors

**DOI:** 10.1186/s13104-018-3678-x

**Published:** 2018-08-13

**Authors:** Marrium Asim, Ghazala Mudassir, Atif Ali Hashmi, Mariam Abid, Ahmareen Khalid Sheikh, Hania Naveed, Maryam Habib, Muhammad Muzzammil Edhi, Amir Khan

**Affiliations:** 1 Pakistan Institute of Medical Sciences, Islamabad, Pakistan; 2 Shifa College of Medicine, Islamabad, Pakistan; 3 Liaquat National Hospital and Medical College, Karachi, Pakistan; 4 Brown University, Providence, RI USA; 5 Kandahar University, Kandahar, Afghanistan

**Keywords:** Fine needle aspiration, Malignant small round cell tumors, Wilms tumor, Lymphoma, Neuroblastoma, Rhabdomyosarcoma

## Abstract

**Objective:**

Fine needle aspiration biospy (FNAB) is a simple, cost effective procedure, which can be carried out in the out-patient department. The objective of our study was to determine the diagnostic accuracy of fine needle aspiration biopsy in small round cell tumors of childhood, keeping histopathology as the gold standard.

**Results:**

Out of these 50 cases, 35 (70%) were small round cell tumors and 15 (30%) cases of other childhood malignancies and certain reactive conditions. In our study, the most common malignant small round cell tumor (SRCT) on histopathology was Wilms tumor (10 cases) followed by non Hodgkin lymphoma (9 cases). FNAB results were correlated with the histological findings and the diagnostic accuracy of SRCT came out to be 98%. The sensitivity and specificity of FNAB in diagnosing SRCT was 97% and 100% respectively. FNAB was found to be a very useful technique in the initial evaluation of any palpable lesion of childhood. Although the small round cell tumors appear cytologically similar, in the hands of experienced cytopathologists, the subtle morphological features can help towards the final diagnosis. In addition, clinical and radiological findings are invaluable assets, which help to reach the final diagnosis.

**Electronic supplementary material:**

The online version of this article (10.1186/s13104-018-3678-x) contains supplementary material, which is available to authorized users.

## Introduction

Small round cell tumors (SRCT) of childhood are a group of tumors, characterized by small round undifferentiated cells, having a resemblance to primitive fetal/embryonal cells. Due to their undifferentiated nature, it is usually difficult to diagnose the specific entities precisely. Fine needle aspiration biopsy is a diagnostic technique in which tissue is obtained for cytological examination from a mass located in any region of the body [1]. It is a low cost, efficient and safe procedure which is usually done on an outpatient basis [2].

FNAB offers a quick diagnosis to the clinician, in turn, contributing to the early management of the patient. Other benefits of FNAB include identification of benign lesions, recognition of malignant tumors thereby helping the surgeon to plan surgery, providing material for immunocytochemistry, electron microscopy and DNA analysis. The main advantages of FNAB over needle biopsy are that there are no contraindications to this procedure and that it can be done quickly as a safe and simple procedure in an office setting. Moreover, it is more cost effective and much clinical expertise is needed [3–5]. The objective of this study was to determine the diagnostic accuracy of FNAB in small round cell tumors of childhood, keeping histopathology as the gold standard.

## Main text

### Methods

The study was conducted in Department of Histopathology, Pakistan Institute of Medical Sciences, Islamabad over a period of one and a half year from 1st January 2016 till 31st July 2017. Children aged 15 years and younger, presenting with palpable lymph node or abdominal/mediastinal mass or soft tissue swelling or a bony lesion were included in the study.

Approval was taken from the hospital ethical committee before the start of the study. All the patients fulfilling the inclusion criteria with suspicion of malignancy referred to the department of pathology for performance of FNAB. The procedure of FNAB was performed according to standard guidelines and slides were made.

The surgical specimens received were processed for H&E and immunohistochemistry was performed where necessary. The slides of FNAB and surgical specimens were examined by two different experienced histopathologists blinded with each other’s interpretation.

The final histopathological diagnosis was correlated with cytological diagnosis and was classed as: complete correlation, correlation with category only and lack of correlation. The correlation was labeled as ‘Complete’ when the specific cytological diagnosis exactly matched the specific histological diagnosis. It was labeled, ‘Correlation with category only’, when cytology was only able to diagnose the lesion as small round blue cell tumor without pinpointing a specific diagnosis. Finally, it was labeled as ‘Lack of correlation’ when cytological and histological diagnosis showed a complete disparity.

Statistical package for social sciences (SPSS 21) was used for data compilation and analysis. Mean and standard deviation were calculated for quantitative variables. Frequency and percentage were calculated for qualitative variables. Chi square was applied to determine association. P-value ≤ 0.05 was taken as significant.

### Results

Age distribution of the patients ranged from 2 months to 15 years with the mean age being 7.38 ± 4.57 years. Of the 50 cases included in the study, 30 (60%) were males and 20 (40%) were females with a male to female ratio being 1.5:1. Descriptive characteristics are given in Table 1.

**Table 1:** Descriptive characteristics of studied population

Characteristic	Frequency (%)
*Age*	
Mean	7.38 ± 4.57 years
*Age groups* (years)	
0–5	23 (46%)
6–10	13 (26%)
11–15	14 (28%)
*Gender*	
Male	30 (60%)
Female	20 (40%)
*Primary complaint*	
Head and neck swelling	22 (44%)
Palpable abdominal mass	17 (34%)
Swelling lower limb	6 (12%
Swelling upper limb	3 (6%)
Inguinal mass	1 (2%)
Sacrococcygeal mass	1 (2%)
*Size* (cm)	
Mean	5.73 ± 3.14
Range	1–15
*Histopathological diagnosis*	
Wilms tumor	10 (20%)
Non-Hodgkin’s lymphoma	9 (18%)
Rhabdomyosarcoma	3 (6%)
Neuroblastoma	4 (8%)
Osteosarcoma	2 (4%)
Small round cell tumors (unclassified)	7 (14%)
Others	15 (30%)

Of all the cases, there were a total of 47 (94%) malignant cases and 3 (6%) benign cases. Among these 50 cases, 35 were small round cell tumors. Wilms tumor was the most commonly diagnosed tumor on histopathology, followed by non Hodgkin lymphoma in 10 (20%) and 9 (18%) cases respectively. In 7 (14%) cases, a diagnosis of SRCT was given, as further characterization into a specific histologic type was not possible. In most cases, the histopathological diagnoses were rendered on routine H&E staining. However in 7 cases, immunohistochemistry was used for confirmation of diagnosis. Of these 7 cases, three were rhabdomyosarcomas, all of which showed desmin and myogenin positivity. Two of these 7 cases were neuroblastomas, positive for chromogranin and synaptophysin. One of the remaining two cases was diagnosed on immunohistochemistry as Ewing sarcoma and the other, synovial sarcoma, showing positivity for CD 99, EMA and Bcl 2.

Thirty-four cases (68%) showed the small round cell tumor morphology and remaining 16 (32%) cases showed variable morphology on FNAB. Amongst the 34 cases of SRCT, the most common diagnosis on FNAB was non Hodgkin lymphoma in 12 (24%) cases, followed by 9 (18%) cases of Wilms tumor. In 8 cases a definitive diagnosis could not be suggested and a final morphological diagnosis of small round cell tumor was given. In one case, the FNAB was non diagnostic. In this case the final diagnosis on histopathology was Wilms tumor.

A strong correlation was found between the diagnosis made by fine needle aspiration biopsy and the final histopathological diagnosis (Table 2). Of the 35 cases of SRCT diagnosed on histopathology, complete correlation was seen in 24 (68.6%) cases and there was lack of correlation in just one case.

**Table 2:** Correlation of FNAB with histopathology of SRCT

Diagnosis	TP	FP	FN	TN	Sensitivity (%)	Specificity (%)	PPV (%)	NPV (%)	Overall accuracy (%)
SRCT (n = 35)	34	0	1	15	97	100	100	93.75	98

The overall diagnostic accuracy of FNAB in diagnosing small round cell tumors was 98%. The sensitivity and specificity of FNAB were 97% and 100% respectively. The PPV and NPV of FNAB were found to be 100% and 93.75% respectively.

The sensitivity and specificity of FNAB in diagnosing individual tumors were showed in Table 3.

**Table 3:** Correlation of FNAB with histopathology of specifically categorized SRCT

Diagnosis	TP	FP	FN	TN	Sensitivity (%)	Specificity (%)	PPV (%)	NPV (%)	Overall accuracy (%)
WT (n = 10)	9	0	1	25	90	100	100	96.1	97.1
NHL (N = 9)	9	3	0	23	100	88.5	75	100	91.4
Neuroblastoma (n = 4)	2	0	2	31	50	100	100	93.9	94.3
Rhabdomyosarcoma (n = 3)	3	0	0	32	100	100	100	100	100

### Discussion

The aim of this study was to determine the diagnostic accuracy of fine needle aspiration biopsy in diagnosing small round cell tumors of childhood. By examining the results, we found FNAB to be a highly sensitive and specific test for the initial diagnosis of tumors with small round cell morphology.

There were a total of fifty cases, of which 35 cases were confirmed as SRCT on histopathology. FNAB was positive for SRCT morphology in 34 of these cases. One case was negative on FNAB which was due to hemorrhagic inadequate aspiration. The reason for high sensitivity and specificity of FNAB in diagnosing SRCT, which in our study came out to be 97% and 100% respectively, is mainly due to repetition of the procedure in case the smear was hemorrhagic or had a low cellularity making definitive diagnosis impossible. A sensitivity of 97.2% and specificity of 81.2% was found for FNAB in a study done by Giramizadeh et al. The diagnostic accuracy was also comparable with our study, i.e. 92.4% [6].

Wilms tumor came out to be the most common malignancy of childhood in our study. Studies done by Razack et al. and Hasan et al. also showed similar results [7, 8]. However this is in sharp contrast with the results of a study done by Maheshwari et al. in which lymphoma was found to be the most common malignant solid tumor [2]. On epidemiological survey of childhood cancer, Stiller found that leukemia was the most common malignancy of childhood followed by lymphoma and central nervous system tumors [9]. In our study, there were 10 cases of Wilms tumor on histopathology of which 9 were correctly recognized as Wilms tumor on FNAB. All the patients had presented with a palpable abdominal mass which was noted by the parents. One case was missed on FNAB, as the aspiration was hemorrhagic and therefore inadequate for diagnosis. The remaining 9 cases showed variable admixture of blastema (Additional file 1: Figure S1a) and mesenchymal elements. In few cases epithelial differentiation was also identified (Additional file 1: Figure S1b). The diagnostic accuracy in our study was high, i.e. 97.1%, which is comparable to other studies. A study carried out by Maheshwari et al. on over 500 patients showed comparable diagnostic accuracy of 92.3% [2]. A study by Fernandez-Pineda et al found FNAB to be a very valuable technique to confirm the diagnosis of Wilms tumor. The sensitivity and specificity of FNAB was found to be 95% and 83.3% respectively [10].

There were 9 histologically confirmed cases of non-Hodgkin lymphoma all of which were correctly identified as such on FNAB (Additional file 1: Figure S1c). The diagnostic accuracy of FNAB in diagnosing non Hodgkin lymphoma in our study was 91.4% which is slightly lower but still comparable to the study done by Alam et al. in which it was 93.3% [11]. Three cases were misclassified on FNAB as lymphomas, amongst which two cases turned out to be neuroblastomas and one case did not show any sign of differentiation on histopathology and was finally diagnosed as small round cell tumor. The results of immunohistochemistry were available in all these three cases. The first two cases were confirmed as neuroblastomas, while the third turned out to be poorly differentiated synovial sarcoma, as it was positive for CD99, EMA and Bcl2.

There were four histologically confirmed cases of neuroblastoma, of which two were correctly identified on FNAB. Both these correctly identified cases presented with a palpable abdominal mass. In both the cases, the CT scan and clinical findings were highly suggestive of neuroblastoma. The FNAB in these two cases showed Homer Wright rosettes (Additional file 1: Figure S1d) and the histology was also characteristic, showing neuropil (Additional file 2: Figure S2a) and ganglion cell differentiation. The other two cases had an atypical presentation with swelling in the cervical region and were diagnosed as small round cell tumor with features suggestive of non Hodgkin lymphoma on FNAB. The histopathology of one of these cases showed neuropil in the background while the other case showed the undifferentiated small round cell morphology. Both these cases were positive for chromogranin and synaptophysin immunohistochemically. The diagnostic accuracy in our study (94.3%) was comparable with a study carried out by Giramizadeh et al. (100%) [6].

There were three histologically confirmed cases of rhabdomyosarcoma, which were all picked up on FNAB correctly. In all these three cases there was a strong clinical as well as radiological suspicion of rhabdomyosarcoma. Two patients had presented with large cheek swelling with widespread local invasion. The third patient presented with a palpable mass in the abdomen. The aspiration cytology showed scattered strap cells and rhabdoid cells in addition to the round blue cells, characteristic of rhabdomyosarcoma. The histopathology was also characteristic (Additional file 2: Figure S2b, c) Two cases were diagnosed as embryonal rhabdomyosarcoma and the third as alveolar rhabdomyosarcoma. All the three cases were positive for desmin and myogenin. Klijanienko et al. in their study found that a good percentage of rhabdomyosarcomas (97.8%) can be correctly identified as such, or as small round cell tumor on FNAB [12].

There were two histologically confirmed cases of osteosarcoma as they featured osteoid production. Both the cases were diagnosed as small round cell tumor on FNAB (Additional file 2: Figure S2d). The characteristic osteoid production was not identified on aspiration cytology which is the most significant limitation of FNAB, as dense collagen cannot be differentiated from osteoid. FNAB is a useful tool only in the multidisciplinary setting, when there is full knowledge of the clinical and radiological findings [13].

On histopathology, the small round cell tumor—unclassified category, comprised 7 cases. One of these cases was diagnosed; yolk sac tumor on histopathology. The FNAB of this case showed a tumor with small round cell morphology, however in view of the clinical information (young child with palpable abdominal mass and raised alpha fetoprotein levels), a final diagnosis “suggestive of yolk sac tumor” was rendered. Germ cell tumors can occasionally appear as small round cell tumors. The other six cases did not show any signs of differentiation on histopathology and were diagnosed as small round cell tumor. Two cases were immunohistochemically confirmed as Ewing sarcoma and synovial sarcoma (which was diagnosed as non-Hodgkin lymphoma on FNAB, as described earlier).

Molecular diagnostic testing of cancers especially next generation sequencing is now becoming routine in many cancers especially lung and breast tumors. Many investigators have suggested that adequacy rate of cytologic material is comparable to that of core biopsy and provide higher quality DNA compared to formalin fixed tissue. Therefore, cytologic material is a valuable source of molecular diagnostic testing in cancers [14].

## Limitations

The main limitation of our study was limited number of cases, however, the study demonstrates that fine needle aspiration biopsy in paediatric tumors, is a useful technique for initial evaluation of any suspicious swelling. It is a very simple, safe and cost effective procedure which can be carried out on OPD basis.

## Additional files

**Additional file 1: Figure S1.** A) FNAB in a case of Wilms tumor showing sheets of blastema cells (H&E ×400). B) FNAB in a case of Wilms tumor showing epithelial differentiation in the form of tubule formation and focal spindle shaped mesenchymal cells (H&E ×100, C) FNAB of cervical lymph node showing sheets of atypical lymphoid cells (H&E ×400). D) A case of neuroblastoma showing formation of rosettes with central neurofibrillary material (H&E ×100).

**Additional file 2: Figure S2.** A) Trucut biopsy of an abdominal mass showing scattered neuroblasts in a background of abundant neuropil (H&E ×400). B) A case of rhabdomyosarcoma showing small round cells, with some cells showing eccentric nuclei and eosinophilic cytoplasm (H&E ×400). C) Biopsy of swelling cheek showing a tumor with an alveolar pattern of growth, diagnosed as alveolar rhabdomyosarcoma (H&E ×200), D) FNAB of knee swelling showing aggregates of small round cells, diagnosed as osteosarcoma on histopathology (H&E ×400).
