# Supplementary figures and images for: Diagnostic accuracy of fine needle aspiration biopsy in pediatric small round cell tumors
Source: BMC Res Notes. 2018 Aug 13;11:573. doi: 10.1186/s13104-018-3678-x (PMC6090781; doi:10.1186/s13104-018-3678-x)

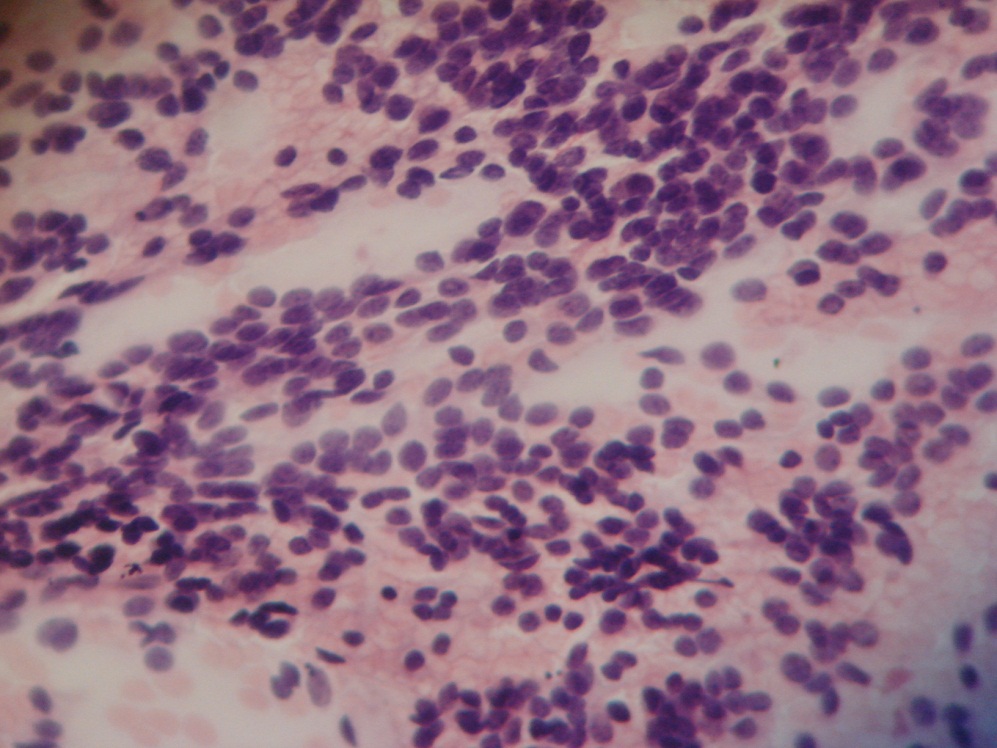

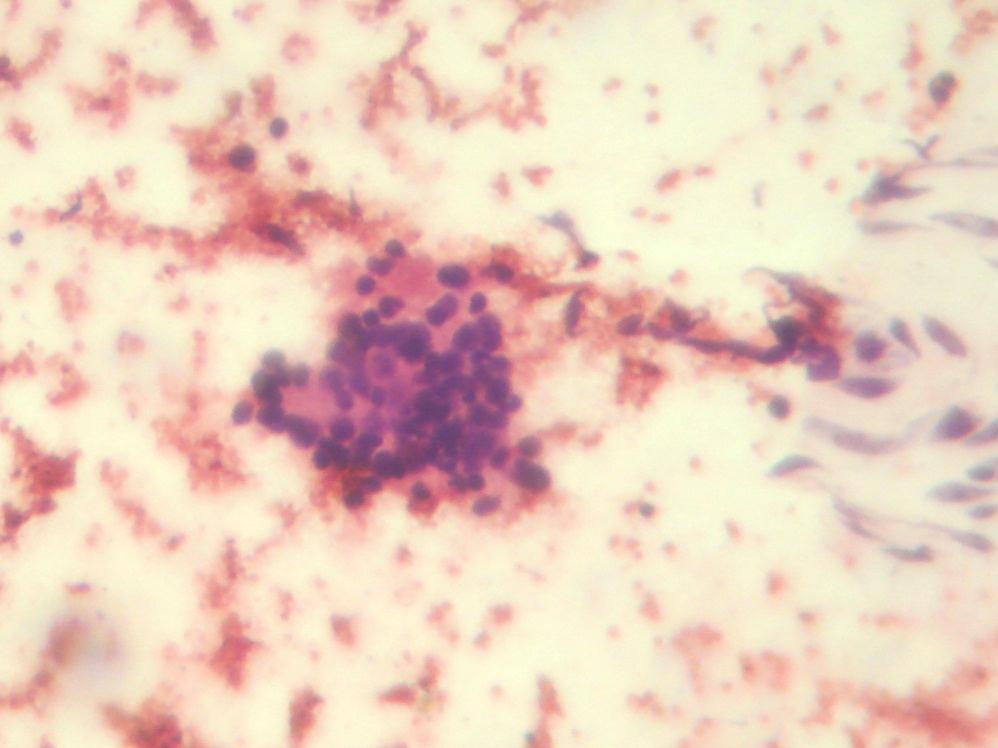


A)

B)


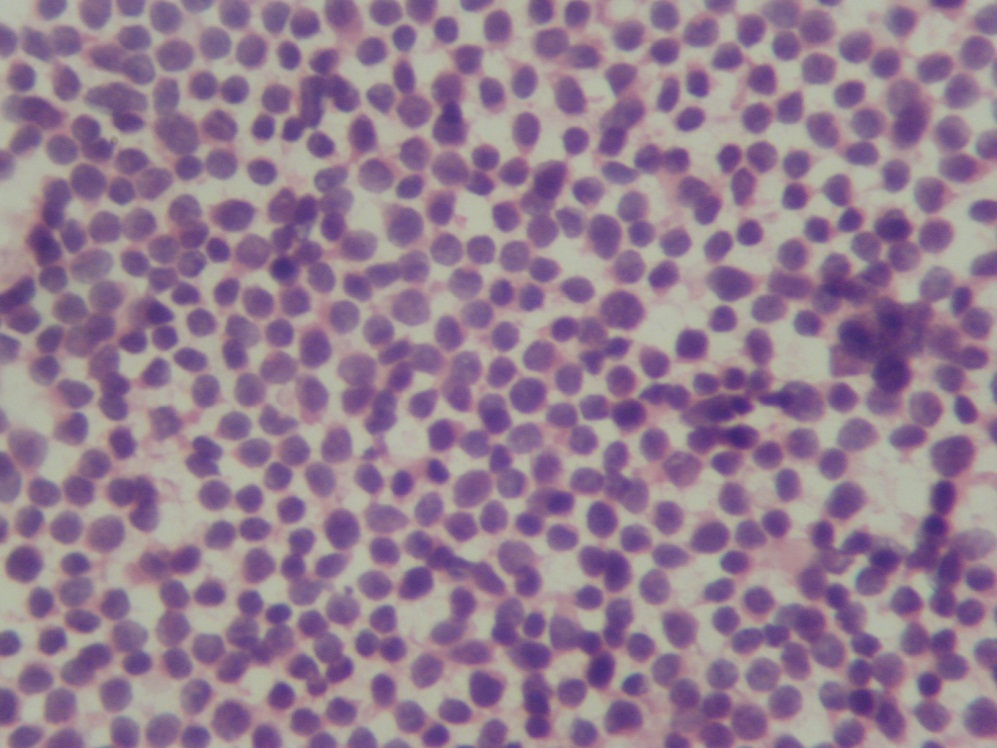

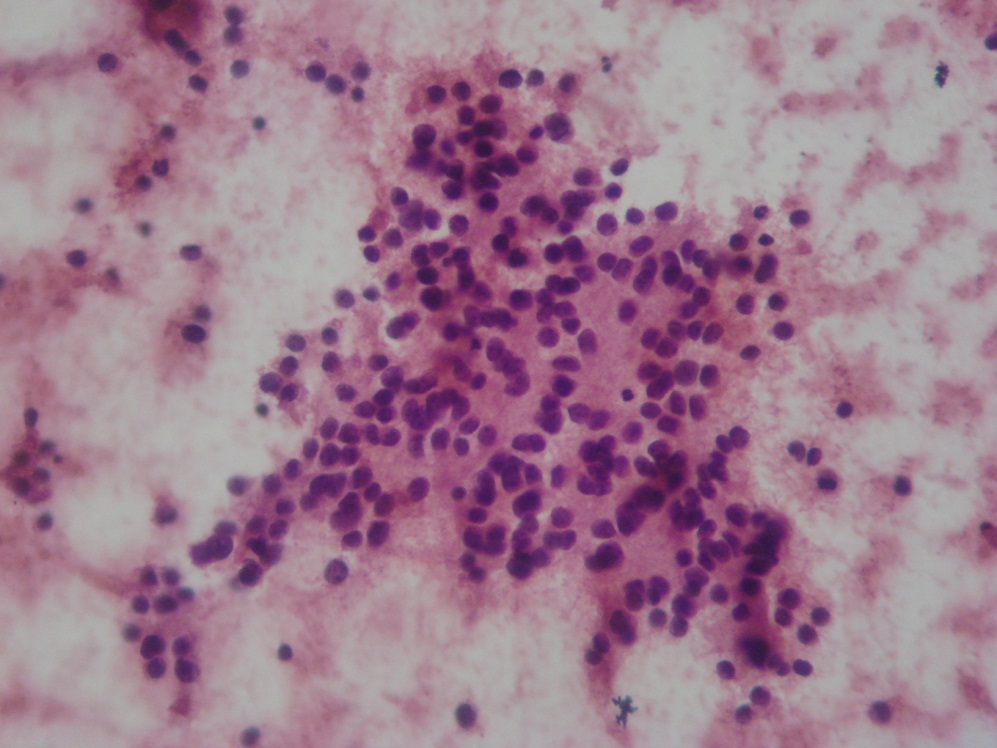


D)

C)

Supplement: Supplementary file 1 — Additional file 1: Figure S1. A) FNAB in a case of Wilms tumor showing sheets of blastema cells (H&E ×400). B) FNAB in a case of Wilms tumor showing epithelial differentiation in the form of tubule formation and focal spindle shaped mesenchymal cells (H&E ×100, C) FNAB of cervical lymph node showing sheets of atypical lymphoid cells (H&E ×400). D) A case of neuroblastoma showing formation of rosettes with central neurofibrillary material (H&E ×100). [file 13104_2018_3678_MOESM1_ESM.doc]

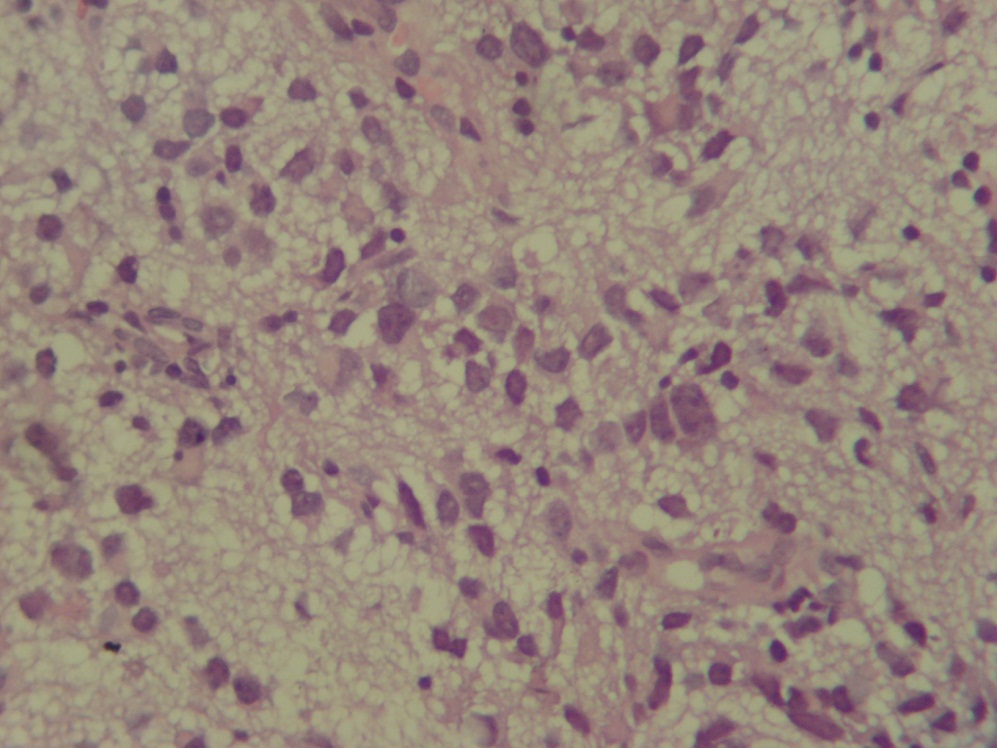

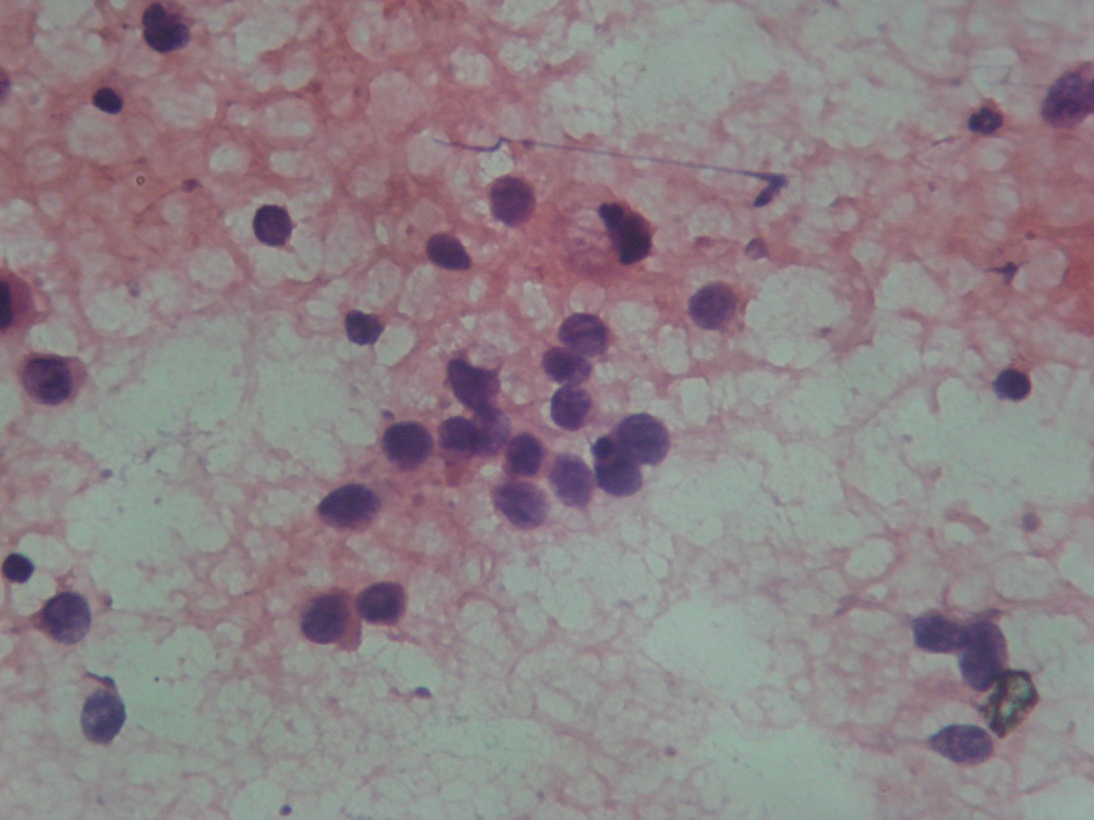

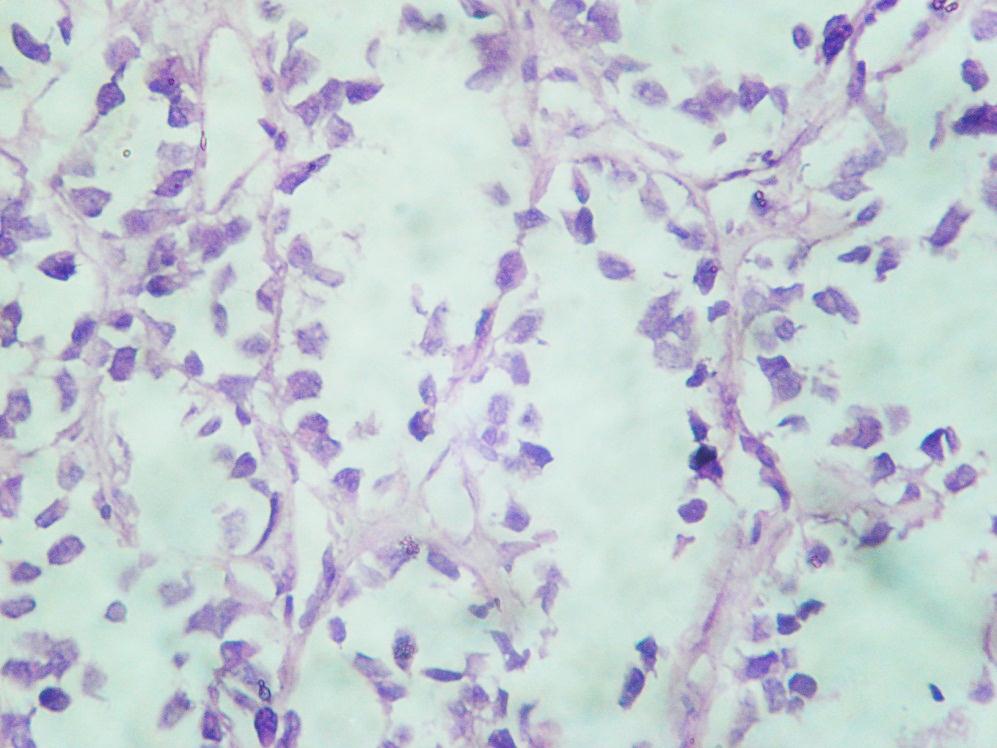

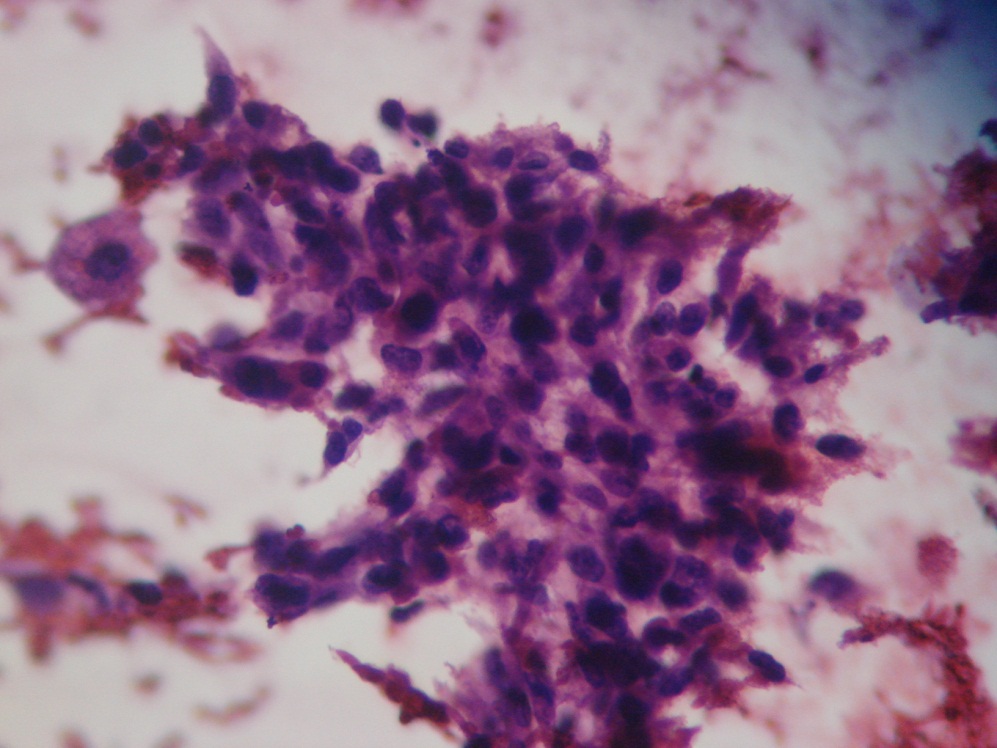


A)

D)

C)

B)

Supplement: Supplementary file 2 — Additional file 2: Figure S2. A) Trucut biopsy of an abdominal mass showing scattered neuroblasts in a background of abundant neuropil (H&E ×400). B) A case of rhabdomyosarcoma showing small round cells, with some cells showing eccentric nuclei and eosinophilic cytoplasm (H&E ×400). C) Biopsy of swelling cheek showing a tumor with an alveolar pattern of growth, diagnosed as alveolar rhabdomyosarcoma (H&E ×200), D) FNAB of knee swelling showing aggregates of small round cells, diagnosed as osteosarcoma on histopathology (H&E ×400). [file 13104_2018_3678_MOESM2_ESM.doc]
